# Comparison of Risk Factors for Erectile Dysfunction (ED) in Type 2 Diabetics and Nondiabetics: A Retrospective Observational Study

**DOI:** 10.7759/cureus.44576

**Published:** 2023-09-02

**Authors:** Ritesh Kumar, Ujwal Kumar, Sameer Trivedi

**Affiliations:** 1 Department of Endocrinology, Diabetes and Metabolism, Institute of Medical Sciences, Banaras Hindu University, Varanasi, IND; 2 Department of Urology, Institute of Medical Sciences, Banaras Hindu University, Varanasi, IND

**Keywords:** erection, obesity, hypertension, diabetes mellitus, erectile dysfunction

## Abstract

Background and aims: We aim to compare the various risk factors for erectile dysfunction (ED) in type 2 diabetes mellitus (DM) and nondiabetic patients.

Materials and methods: We retrospectively collected and evaluated the data of 175 OPD patients with ED. We included 138 patients of ED from endocrinology and urology OPD after exclusion. ED was assessed by using a questionnaire adapted from the abridged five-item version of the International Index of Erectile Function (IIEF-5) score.

Results: A total of 96 (69.56%) were diabetic, and 42 (30.43%) were nondiabetic. The majority of patients (62.31 %) were in the age group of 40-60 years. Thirty-nine (28.26%) were alcoholics, and 55 (40%) were smokers. The average duration of diabetes was 6.6±1.5 years. Hypertension was present in 49 (35.5%). Diabetic patients were significantly older (47.9±8.2 vs. 40.2±7.6 years, p=0.0001) and obese (BMI (kg/m^2^), 27.3±5.4 vs. 24.6±3.9, p=0.004). Waist circumference in diabetics was 95.3±10.9, as compared to nondiabetics, which was 89.6±9.2 cm (p=0.0037). The IIEF-5 score was significantly lower in diabetic subjects in comparison to nondiabetics (9.4±3.2 vs. 12.1±3.6 p=0.0001). Moderate-to-severe ED was more common in diabetic patients (76%) in comparison to nondiabetics (59.5%). The prevalence of mild and mild-to-moderate ED was 11.45 % and 12.5 % in diabetic patients in comparison to 16.7% and 23.5% in nondiabetics, respectively. The prevalence of hypertension and coronary artery was higher in diabetics in comparison with nondiabetics. Hypertension was significantly higher in diabetic patients with ED (42.7% vs. 19.04%, p=0.0075), but coronary artery disease was not statistically significant (8.3% vs. 2.3%, p=0.1925). LH (2.6±0.7 vs. 3.5±0, p=0.0001) and testosterone (312.1±110.7 vs. 367.8±115.1, p=0.0081) were significantly lower in diabetics in comparison to nondiabetics.

Conclusions: The IIEF-5 score was lower in diabetic cases as compared to those without diabetes. The factors that significantly contributed to ED in type 2 DM patients, as compared to nondiabetic patients, were age, BMI, waist circumference, hypertension, poor glycemic control, LH, and testosterone levels.

## Introduction

Erectile dysfunction (ED) is defined as the consistent and recurrent inability to have sufficient erection to engage in satisfactory sexual intercourse, leading to decreased quality of life of men and their partners along with stress [1]. ED is assessed by clinical history taking and assessment of various risk factors, such as hypertension, diabetes mellitus (DM), coronary artery disease, and hyperlipidemia for ED, and the use of the International Index of Erectile Function (IIEF-5) questionnaire. In previous studies, the prevalence of ED has been shown to range from 2% to 9% in men between the ages of 40 and 49 years, which increases to 20-40% in men aged 60-69 years. In men older than 70 years, the prevalence of ED ranges from 50% to 100% [2]. In recent studies, ED has been reported with increased incidence in men less than 40 years of age [3,4]. Studies have shown that about 87% of young men with ED have an organic cause either vascular, hormonal, medication-induced, neurological, or fibroproliferative [5]. DM leads to neuropathy, macroangiopathy, and microangiopathy in the long run. DM leads to sexual dysfunction in both women and men with ED being the most important dysfunction in men with DM [6]. ED was found in 67% and 71% of patients with diabetes and hypertension, respectively, in a study conducted using the 11EF-5 [7]. Sexual dysfunction in diabetics is due to coexisting hypertension, obesity, metabolic syndrome, smoking, and atherogenic dyslipidemia [8]. ED is itself a marker of significantly increased risk of coronary artery disease, cardiovascular disease, and stroke [9]. ED can be the presenting symptom of diabetes in some patients, especially among elderly patients. Some studies have shown that diabetes is often diagnosed for patients seeking help for ED [10]. In type 2 DM patients, ED occurs about 10-15 years earlier, and the prevalence of ED in these patients is about 1.9-4 times higher as compared to those without diabetes [10-12]. There are limited studies about factors contributing to ED in India, and to the best of our knowledge, no study is available to compare the various parameters of ED in diabetic and nondiabetic patients. Our study’s objective is to compare the various risk factors for ED in type 2 DM patients and those without DM and to see if the risk factors are significant between the two groups.

## Materials and methods

In this retrospective observational study, data of patients reporting with ED to the Endocrinology & Urology Out-Patient Department (OPD), Institute of Medical Sciences, Banaras Hindu University, Varanasi, between January 2020 and December 2022 were collected. Patients were divided into two groups: one having type 2 DM and the other that were nondiabetics. Patients with ages below 18 years and above 70 years were excluded. Secondary ED from genetic, neurological, or surgical causes were also excluded from the study. Type 1 DM, micropenis, and/or disorder of sex development are also excluded. Sociodemographics, clinical parameters, and laboratory findings were collected from OPD records. DM was diagnosed based on a 75-g oral glucose tolerance test (OGTT) and/or glycosylated hemoglobin (HbA1c) or based on records in OPD tickets for previously diagnosed diabetic cases. All patients with ED were evaluated for age, sex, height, weight, and body mass index (BMI). A stadiometer with a head held in the Frankfurt plane to the nearest 0.1 cm was used for recording height (in cm). Weight was measured (in kg) using a beam balance to the nearest 0.1 kg. Calculation of BMI was done using the formula weight in kilograms (kg) divided by height in meters squared (kg/m2). Waist circumference was measured at the umbilical level in a standing position at minimal respiration and rounded off to the nearest 0.1 cm.

The chemiluminescent immunoassay method by using a commercial kit (Access LH kit; Beckman Coulter, Fullerton, CA, USA) was used for assaying luteinizing hormone (LH). The inter- and intra-assay coefficients of variation (CV) were <6.4% and <4.3%, respectively, and sensitivity was 0.2 mIU/mL. Testosterone assay was done by chemiluminescent immunoassay (CLIA, UniCell DXI 800; Access testosterone; Beckman Coulter Fullerton, CA, USA)). Inter-assay CV was 5.4% for 26 nmol/L, 3.9% for 16.6 nmol/L, and 7.1% for 2 nmol/L; intra-assay CV was 2.6% for 29.4 nmol/L, 2.3% for 16.6 nmol/L, and 4.3% for 2 nmol/L, total imprecision <10% at a concentration between 6.9 and 34.7 nmol/L, and lower limit of sensitivity 0.35 nmol/L. The HbA1c assay was done using ionic exchange high-pressure liquid chromatography (HPLC) on a D-10 hemoglobin testing system (Bio-Rad Laboratories; Hercules, California, USA). Enzymatically blood glucose was measured using the glucose oxidase peroxidase method. Automated Mindray SAL 6000 (Shenzhen, China) was used for other blood investigations.

ED was assessed by using a questionnaire adapted from the abridged five-item version of the IIEF-5 score. The ED was classified based on the scores of the IIEF-5. Severe ED: Study participants who scored 5-7 out of 25 points. Moderate ED: Study participants who scored 8-11 out of 25 points. Mild-to-moderate ED: Study participants who scored 12-16 out of 25 points. Mild ED: Study participants who scored 17-21 out of 25 points. CAD is a condition of atherosclerotic occlusion of coronary arteries, leading to ischemia of the myocardium. CAD was diagnosed on the basis of ECG (ST and T wave changes or presence of pathological Q wave), 2D echocardiography (presence of residual wall motion abnormality or left ventricular diastolic dysfunction), or angiography finding (occlusion more than 50%). Further, patients having a history of angioplasty or coronary artery bypass graft surgery or patients taking medications for CAD based on OPD records were labeled as having CAD.

Quantitative variables were expressed as mean±standard deviation and analyzed using the independent sample t-test. Qualitative variables were expressed as percentages and were analyzed using the Fischer exact test. A paired t-test was used to compare the IIEF-5 scores from baseline to after treatment with tadalafil. A one-way ANOVA test was used for comparing three or more independent groups. Statistical Package for Social Sciences (SPSS) version 25.0 (IBM Corp., Armonk, NY, USA) was used for data analysis, and p-value<0.05 was considered significant.

## Results

**Figure 1:**
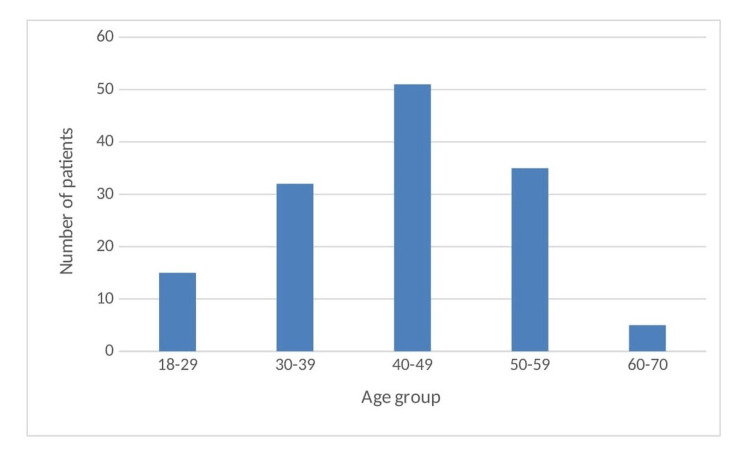
Age distribution of patients

**Figure 2:**
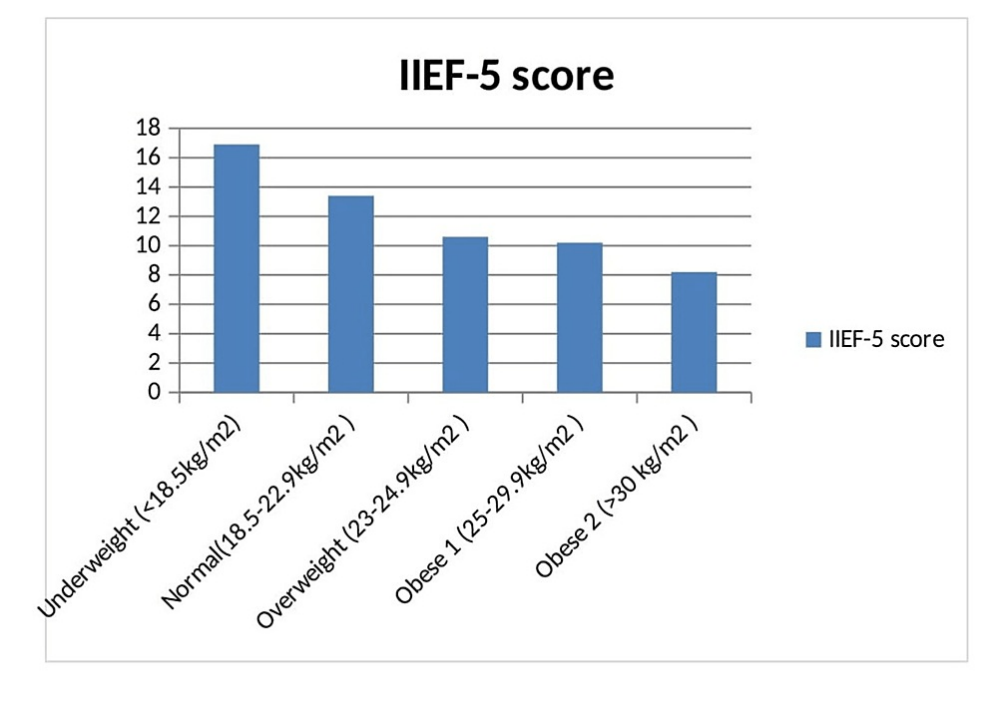
IIEF-5 score of patients according to BMI classification

We retrospectively collected data from 175 OPD patients with ED. ED due to other causes such as primary hypogonadism (n=12), Kalmann syndrome (n=13), a disorder of sex development (n=5), and pelvis surgery (n=7) were excluded. Baseline characteristics are given in Table number 1. A total of 96 (69.56%) had type 2 DM, and 42 (30.43%) were nondiabetics. The majority of patients (62.31 %) were in the age group of 40-60 years (Table 1, Figure 1 ). Thirty-nine (28.26%) were alcoholics, and 55 patients (39.9%) were smokers. The average duration of diabetes was 6.6±1.5 years. Hypertension was present in 49 (35.5%) patients. Diabetic patients were significantly older (47.9±8.2 vs. 40.2±7.6 years, p=0.0001) and obese (BMI (kg/m2) 27.3±5.4 vs. 24.6±3.9, p=0.004). Waist circumference in diabetics was 95.3±10.9, as compared to nondiabetics in which it was 89.6± 9.2 cm, p=0.0037. The IIEF-5 score was significantly lower in diabetic subjects in comparison to nondiabetics (9.4±3.2 vs. 12.1±3.6, p=0.0001) (Table 2). Moderate-to-severe ED was more common in diabetic patients (76%) in comparison to nondiabetics (59.5%). The prevalence of mild and mild-to-moderate ED was 11.45 % and 12.5% in diabetic patients in comparison to 16.7% and 23.5% in nondiabetics, respectively. The prevalence of hypertension and coronary artery was higher in diabetics in comparison with nondiabetics. Hypertension was significantly higher in diabetic patients with ED (42.7% vs. 19.04%, p=0.0075), but coronary artery disease was not statistically significant (8.3% vs. 2.3%, p=0.1925). LH (2.6±0.7 vs. 3.5±0.9, p=0.0001) and testosterone (312.1±110.7 vs. 367.8±115.1, p=0.0081) were significantly lower in diabetics in comparison to nondiabetics. On the logistic regression model, we did not find smoking (p=0.087), alcohol (p=0.131), CAD (p=0.092), LDL (p=0.236), and GFR (p=0.099) as independent predictors for ED because of almost equal distribution between the two groups of DM and nondiabetic patients. Follow-up records of the IIEF-5 scores were available in only 86 patients (89.6%) in diabetic patients and in 38 (90.5 %) in nondiabetic patients. The average duration of follow-up was 3±1.5 months. There was a significant improvement in the IIEF-5 scores after treatment in both diabetics (9.4±3.2 vs. 15.7±5.9, p=0.0001) and nondiabetics (12.1±3.6 vs. 20.4±6.1, p=0.0001) with tadalafil. In our study, 28.3% of our patients were overweight, 31.9% had obesity grade 1, and 17.4% had obesity grade 2, making a total of 77.6% of patients having BMI above the standard limits, and IIEF-5 scores decreased significantly with increasing BMI (Table 3, Figure 2).

**Table 1:** Baseline characteristics of the study population

Total number of patients	138
Age (years)	
18-29	15
30-39	32
40-49	51
50-59	35
60-70	5
Duration of symptoms (years) (mean±SD)	2.5 ± 0.6
Alcohol	
Never	99 (71.7%)
Current/former	39 (28.3%)
Smokers	
Never	83 (60.1%)
Current/former	55 (39.9%)
Diabetic/nondiabetic	96 (69.6%)/42 (30.4%)
Duration of diabetes (years) (mean±SD)	6.6 ± 1.5
Hypertension	49 (35.5%)
Coronary artery disease	9 (6.5%)
BMI (kg/m^2^) (mean±SD)	25.7±4.5
Systolic blood pressure (mm of Hg) (mean±SD)	133.8±13.5
Diastolic blood pressure (mm of Hg) (mean±SD)	84.6±6.4
Fasting plasma glucose (mg/dL) (mean±SD)	136.6±39.8
Post prandial plasma glucose (mg/dL) (mean±SD)	202.7±91.7
HbA1C (%) (mean±SD)	7.2±1.2
Total cholesterol (mg/dL) (mean±SD)	137.2±39.6
Triglycerides (mg/dL) (mean±SD)	166.7±81.9
LDL cholesterol (mg/dL) (mean±SD)	81.5±35.4
HDL cholesterol (mg/dL) (mean±SD)	33.2±15.1
VLDL cholesterol (mg/dL) (mean±SD)	33.6±19.5
GFR (mL/minute/1.73 m^2^) (mean±SD)	87.5±30.7
LH (IU/mL) (mean±SD)	2.9±0.8
Testosterone (ng/dL) (mean±SD)	325.7±112.5

**Table 2:** Difference in various factors in diabetics versus nondiabetics

	Diabetics (n=96)	Nondiabetics (n=42)	P-value
Age	47.9±8.2	40.2±7.6	0.0001
BMI(Kg/m^2 ^)	27.3±5.4	24.6±3.9	0.004
Waist circumference	95.3± 10.9	89.6± 9.2	0.0037
IIEF-5 score	9.4±3.2	12.1±3.6	0.0001
IIEF-5 grading			0.1465
Mild (17-21)	11 (11.4%)	7 (16.7%)	
Mild to moderate (12-16)	12 (12.5%)	10 (23.8%)	
Moderate (8-11)	29 (30.2%)	14 (33.3%)	
Severe (5-7)	44 (45.8%)	11 (26.2%)	
Hypertension	41 (42.7%)	8 (19.04%)	0.0075
Coronary artery disease	8 (8.3%)	1 (2.3%)	0.1925
Hemoglobin (Gm/ dL) (mean±SD)	10.5 ±2.2	10.9±2.4	0.3409
GFR (mL/minute/1.73 m^2^) (mean±SD)	82.8±30.1	90.3±31.9	0.1882
Fasting plasma glucose (mg/dL) (mean±SD)	157±67.3	90±13.9	0.0001
Post prandial plasma glucose (mg/dL) (mean±SD)	234±101.4	131±60.2	0.0001
HbA1C (%) (mean±SD)	8.9 ±2.1 %	5.8%±0.9	0.0001
LH (IU/mL) (mean±SD)	2.6±0.7	3.5±0.9	0.0001
Testosterone (ng/dL) (mean±SD)	312.1±110.7	367.8±115.1	0.0081
Total cholesterol (mg/dL) (mean±SD)	135 ± 40.1	141.1± 42.3	0.4201
Triglycerides (mg/dL) (mean±SD)	160.5 ± 78.4	174.2±84.8	0.3542
LDL cholesterol (mg/dL) (mean±SD)	80.1± 36.2	82.4±35.7	0.7307
HDL cholesterol (mg/dL) (mean±SD)	32.9± 16.7	33.6±15.4	0.8170
VLDL cholesterol (mg/dL) (mean±SD)	32.1 ±18.9	34.7±20.1	0.4671

**Table 3:** IIEF-5 scores according to BMI classification

BMI classification	Number of patients	IIEF-5 score	P-value
Underweight (<18.5 kg/m^2^)	5 (3.6%)	16.9±3.8	<0.0001
Normal (18.5-22.9 kg/m^2 ^)	26 (18.8%)	13.4±2.1	
Overweight (23-24.9 kg/m^2 ^)	39 (28.3%)	10.6±1.9	
Obese 1 (25-29.9 kg/m^2 ^)	44 (31.9%)	10.2±1.1	
Obese 2 (>30 kg/m^2 ^)	24 (17.4%)	8.2±0.6	

## Discussion

In our study, the prevalence of diabetes in patients with ED was 69.5%. Previous epidemiological studies reported a prevalence of diabetes between 22.2% and 37.6% [8,13,14]. The high prevalence of diabetes in patients with ED was probably due to referral bias (poor response to treatment in diabetic patients and patient data from endocrinology OPD), screening of diabetes by all three methods (FBS, PPBS, and HbA1C), and wide range of age. Skeldon et al. reported that the prevalence of undiagnosed diabetes (based on only fasting plasma sugar) was 11.5% in patients with ED, which increased to 19.1% in middle-aged men (40-59) years old [15]. Previous epidemiological studies have reported a lesser prevalence of diabetes because the diagnosis was made based on history or by doing only one screening test. The average duration of diabetes in our study was 6.6 years. Bacon et al. found that, for men between ages 53-90 years, the risk of ED increased with the duration of type 2 DM to nearly two times, as compared to men who did not have diabetes, and the risk in middle-aged and older men was nearly two times, as compared to men without diabetes [16]. Cardiovascular diseases and DM have been shown to have additive effects on ED across age groups [16]. In our study, the prevalence of hypertension was 35.5%. Similarly, other studies have reported the prevalence of hypertension in ED between 38.4% and 41.6%, respectively [8,13].

In our study, we found that 28.3% of patients had a history of alcohol consumption. ED and alcohol consumption have a complex relationship. Alcohol consumption has been believed to be an aphrodisiac, but alcohol has been considered as a risk factor for ED. A meta-analysis of population-based cross-sectional studies has found significantly decreased odds of ED among alcohol consumers [17]. The prospective Massachusetts Male Aging Study found no significant difference in the incidence of ED among different degrees of alcohol consumption [18]. About 39.9% of our patients had a history of cigarette smoking. Smoking damages the vascular endothelium and impairs eNOS-mediated vasodilatation along with alteration in the elastin of the extracellular matrix and calcification of elastic fibers producing arterial stiffness, leading to ED in smokers. Current smokers have an odds ratio of 1.5-1.8 for ED, with statistically significant confidence intervals, in several studies, and for former smokers, the odds ratio is 1.29 [19,20].

IIEF-5 scores were lower in patients with diabetes in comparison to nondiabetics. Factors that contributed to ED in DM patients were age, BMI, waist circumference, hypertension, poor glycemic control, LH, and testosterone levels. The prevalence of hypertension was significantly higher in diabetics as compared to nondiabetics with ED, but the prevalence of coronary artery disease, though high in the diabetic group, was not statistically significant in the two groups in our study. ED has been shown to be present before the onset of coronary artery disease symptoms and has been considered an early sign of latent ischemic heart disease, and severe ED has been regarded as an independent risk factor for coronary artery disease [21].

In our study, the elderly population was less in number, probably due to less number of patients reporting to OPD for ED in this age group due to social stigma. We found increased BMI and waist circumference in diabetic patients in comparison to nondiabetics. It is well-known that obesity is a risk factor for ED in diabetic patients [22-24]. Subclinical inflammation and oxidative stress in obesity lead to leptin resistance that results in low LH and low testosterone, which is one of the causes of ED [25]. Insulin resistance in obesity elevates oxidative stress and inflammatory cytokines, such as TNF-α and IL-6 in endothelial cells, which decreases nitric oxide bioavailability and induces endothelial dysfunction [26,27].

In our study, 28.3% of our patients were overweight, 31.9% had obesity grade 1, and 17.4% had obesity grade 2, making a total of 77.6% of patients having BMI above the standard limits, and the IIEF-5 scores decreased significantly with increasing BMI. Obesity by decreasing testosterone levels and increased cardiovascular risk acts as a significant risk factor for ED. Studies in the past have shown similar findings and have shown a direct relationship between obesity and ED [28,29].

To the best of our knowledge, this is the only study comparing risk factors for ED in DM patients to nondiabetics. There are certain limitations to our study. It is a single-center retrospective study, and long-term follow-up data were not available. The number of patients in the nondiabetic group was less, and factors such as smoking, alcohol, and CAD had equal percentage distribution between the two groups, so it could not be assessed for significance. Large multicentric prospective studies are needed to compare ED in the two groups.

## Conclusions

The IIEF-5 scores were lower in diabetic cases as compared to those without diabetes. The factors that significantly contributed to ED in type 2 DM patients, as compared to nondiabetic patients, were age, waist circumference, hypertension, poor glycemic control, LH, and testosterone levels. Abnormal BMI was also associated with lower IIEF-5 scores and contributed to ED. Further large prospective studies are needed to compare the various risk factors between diabetic and nondiabetic ED cases.
